# Risk Without Values: Including Indigenous Perspectives in Climate Risk Assessments

**DOI:** 10.1111/risa.70309

**Published:** 2026-07-10

**Authors:** Marcelle Scadden, George W. Warren, Darcy Glenn, Lily George, Tom M. Logan

**Affiliations:** ^1^ Civil & Environmental Engineering University of Canterbury Christchurch New Zealand; ^2^ European Centre for Environment & Human Health (ECEHH) University of Exeter Penryn Campus Penryn Cornwall UK; ^3^ School of Social & Cultural Studies Victoria University of Wellington Wellington New Zealand; ^4^ Urban Intelligence Christchurch New Zealand

**Keywords:** cultural values, Indigenous worldviews, relational risk, risk assessment framework limitations, risk perceptions

## Abstract

Climate risk assessments (CRAs) increasingly acknowledge Indigenous communities as disproportionately exposed to climate change, yet they rarely engage Indigenous perspectives as distinct ways of understanding risk. In a thematic review of CRAs self‐submitted to the Carbon Disclosure Project (CDP) by English‐speaking international cities, we examined how Indigenous perspectives, values, and worldviews are represented. Although many reports recognized unequal exposure, few addressed the structural sources of inequity or differences in how risk, consequence, and adaptation are conceptualized. Indigenous knowledge and sociocultural dimensions were typically incorporated within prevailing technocratic frameworks rather than recognized as alternative risk framings. As a result, Indigenous perspectives were translated into dominant institutional paradigms instead of shaping the conceptual foundations of assessment itself. These findings suggest that procedural inclusion is insufficient. Meaningful engagement requires climate risk frameworks capable of accommodating relational, place‐based, and Indigenous understandings of risk, rather than subsuming them within existing models.

## 1 Introduction

Climate risk assessments (CRAs) shape future planning and risk management, yet what is considered “at risk” depends on sociocultural context. This shapes what people interpret as risk, resulting in different identifications of where the risk is coming from, what is impacted, and adaptation options (Boholm and Corvellec 2011; Boholm 2009). Prevailing approaches rarely account for this diversity, instead applying a largely technocratic framing of risk.

CRAs are part of the initial stages of adaptation planning to prepare communities for climate change, framed as systematic processes formally analyzing the likelihood of future events and their consequences to inform adaptation options and decisions (Hedlund 2023). Often conducted by consultants on behalf of central and local government agencies, technical risk assessors make culturally embedded value judgments according to their institutionalized interpretations of risk and value (Boholm and Corvellec 2011). Values are foundational to worldviews and are shared intergenerationally and culturally, and grow according to experience to further inform value systems (Le Heron et al. 2022; Royal 2003). Practitioners structure the risk problem based on where they see the harm as coming from, decide which consequences to include to which values, and estimate exposures according to their worldviews and the knowledge they have developed in their field (Boholm and Corvellec 2011; Slovic 1999; Aven 2015). The outcome of the technical CRA process is disseminated in a report that is used to communicate the likelihood of hazards and consequences to decision‐makers, including community members (Boholm 2009; Aven 2015). Values are often assumed to be added later in the risk management process by decision‐makers; however, both expert and decision‐maker reviews are “based on a combination of factual and value‐based considerations” (Aven 2015, 3).

Relational theories of risk make explicit the connection between cultural context and social construction of risk, considering the dynamic interactions of the risk object, the objects at risk, and the links between them acknowledged by the observers (Boholm and Corvellec 2011; Hilgartner 1992). Because these objects and links are socioculturally embedded and constitutive, observers with diverse knowledge systems may construct the same phenomenon differently: as a risk, something at risk, perhaps neither, or even an opportunity or benefit instead (Boholm and Corvellec 2011; Rickard 2021). These differences in worldviews and values shape how risks are constructed and perceived (see Dake 1991; Douglas 2002; Douglas and Wildavsky 1982, 1983; Krimsky and Golding 1992; Pidgeon 1998; Rickard 2021; Rippl 2002; Steg and Sievers 2000), influencing which risks are prioritized and how responses are designed (Adger et al. 2012).

Different perspectives are analytically relevant to CRAs when they meet criteria intrinsic to assessing risk, including differential exposure to locally specific climate risks and their consequences, knowledge, and experiences relevant to understanding the context and impacts, and value systems that may have diverging priorities (Anderson et al. 2024; Beck 1996; Whyte 2016). As CRAs necessarily involve judgments related to significance, harm, and acceptable trade‐offs, the perspectives of groups whose livelihoods, lands, and adaptive capacities are directly implicated in assessment outcomes are particularly important to the quality and legitimacy of the process (Bailey‐Winiata et al. 2024).

Indigenous worldviews (the foundational framework of beliefs and values individuals and communities hold) and values (the things we put a value on as well as the ethical and moral principles we hold) are situated in space and time to their interconnected sociocultural contexts and environmental systems (Menzies et al. 2022; Pidgeon 1998; Royal 2003; Stoffle and Arnold 2003; Wale and Huson 2024; Yletyinen et al. 2022). Community environmental relationships have been formed over many generations, providing situated, place‐based context that is both demonstrative of local impacts from global hazards and reflective of the community experiencing those impacts. For example, Numic people (United States of America [USA]) describe their interconnectivity with the environment as informing their views on radioactivity through the “angry rock” concept, derived from accumulated emotions associated with environmental processes, transportation, and mishandling (Stoffle and Arnold 2003). However, since their definition of radioactivity differed from government agencies, during conversations regarding waste facilities, the groups “talked past each other” (Stoffle and Arnold 2003, 237). Similarly, in the Tiwi Islands (Australia), Tiwi Rangers observed changes in their environment, particularly to systems and services they rely on, that were not detected by “Western scientific instruments” (Barnett et al. 2023, 15). Failure to recognize what is valued, especially beyond financial measures, can therefore lead to inappropriate risk calculations, cross‐cultural communication breakdowns, and maladaptive outcomes, as adaptation options will fail to prioritize what matters most to local communities.

When Indigenous values are excluded, risk assessments can misjudge the threats communities face, leading to adaptation strategies that are ineffective and culturally inappropriate (Hoffman and Oliver‐Smith 2002; Lazrus 2015; McNeeley and Lazrus 2014; Wale and Huson 2024). This is especially consequential for Indigenous communities, where colonial structures have imposed socioeconomic and environmental conditions that exacerbate vulnerability and lead to disproportionate risks from climate change, as well as impacted abilities to enact decisions (IPCC 2023; Whyte 2016). However, Indigenous peoples should not be “viewed as victims of the *effects* of climate change” but “as *agents* of environmental conservation” who must be actively involved in processes that affect them (Etchart 2017, 1, author's emphasis).

These issues reflect deeper limitations in prevailing approaches to risk assessment. When values differ across groups, but one viewpoint dominates in embedded colonial structures, the definition of risk determines which adaptation options are prioritized (Slovic 1999). Frameworks designed and applied from a single worldview can, therefore, be ill‐suited to capture the concerns of others; in practice, this often results in Indigenous values being neglected in risk assessment and management (Le Heron et al. 2024). Although cultural theories of risk, such as Douglas’ grid‐group typologies, have been influential (Douglas and Wildavsky 1982, 1983), they have also been critiqued as reductive, deterministic, and insufficient for capturing lived experience and context‐specific understandings of risk (Alaszewski 2015; Boholm 1996, 2015; van der Linden 2016; Wilkinson 2001; McEvoy et al. 2017; Verweij et al. 2006). Addressing these limitations requires approaches that incorporate values from the outset of the risk management process and engage communities as active participants. Participatory and dialogic approaches to risk assessment and communication can improve the relevance, legitimacy, and robustness of risk assessments by integrating place‐based knowledge and diverse value systems (Demeritt and Nobert 2014; McComas et al. 2008; Slovic 1999; Balog‐Way et al. 2020). This is particularly important for transboundary risks such as climate change, which require collaboration across groups with different worldviews, practices, and priorities (Blackett et al. 2024; Boholm 2015; Vaughan 1995).

Despite recognition of the importance of Indigenous perspectives in climate adaptation (Dorji et al. 2024; Simpson et al. 2021; Wale and Huson 2024), it remains unclear how Indigenous communities are currently represented in practice within CRAs. Although policy and academic literature call for more inclusive approaches (Adger et al. 2012; Arteaga et al. 2023; Dorji et al. 2024; Lazrus 2015; McNeeley and Lazrus 2014), there has been no structured, comparative analysis of city‐level CRAs to assess whether, and how, Indigenous values, knowledge systems, and worldviews are incorporated. Without this empirical baseline, it is difficult to evaluate progress toward more culturally legitimate and pluralistic assessment processes.

To address this gap, we analyze city‐level CRAs to examine the extent and manner of Indigenous inclusion and what this reveals about how risk is framed in practice. We conducted a grey literature review of CRAs submitted by cities internationally to the database of the Carbon Disclosure Project (CDP), the most comprehensive publicly available collection of self‐submitted climate adaptation documentation (Sheehan et al. 2022). We focus on city‐level reports due to the availability of data and the relevance of the local level. Rather than comparing cities directly, we assess how Indigenous communities, values, and worldviews are represented within formal CRAs to identify recurring patterns and evaluate the advantages and limitations of current assessment practice.

Specifically, we ask the following:

1. To what extent do city‑level CRAs include Indigenous communities and perspectives?
2. How are Indigenous worldviews and values represented in these assessments?
3. What do these patterns reveal about the advantages and limitations of current frameworks and the potential need for more relational or pluralistic approaches to risk assessment?

## 2 Methods

**FIGURE 1:**
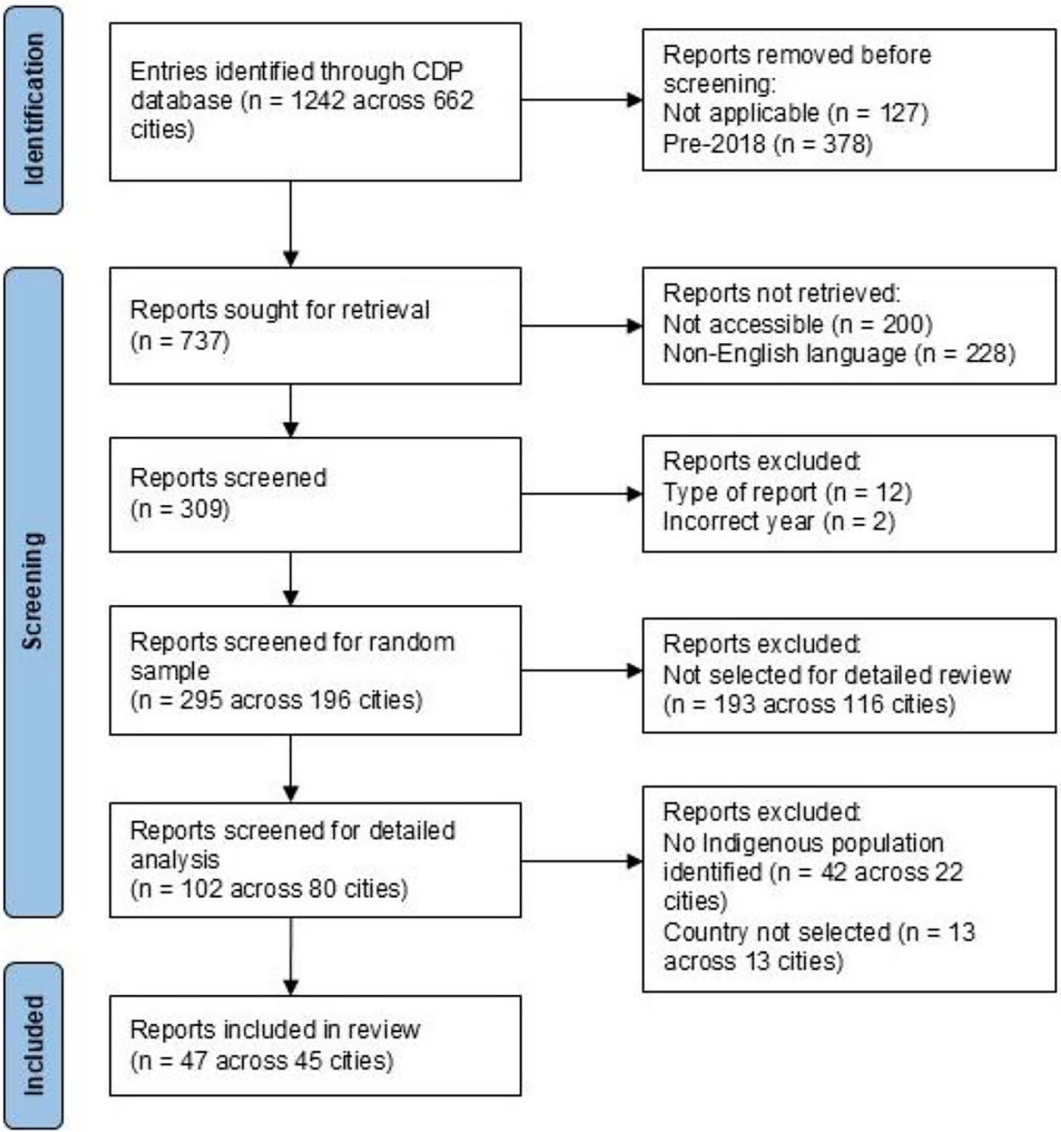
Selection criteria for the CDP climate risk assessment reports following the PRISMA flow diagram (Page et al. 2021). CDP, Carbon Disclosure Project.

In order to explore how CRAs and adaptation plans have been framed and what has been included across a range of factors, we conducted a thematic review of grey literature using multi‐stage purposive sampling between April and May 2024. The first stage involved reviewing climate risk and adaptation reports self‐submitted to the CDP database (CDP 2022 Cities Climate Risk and Vulnerability Assessments) collected in partnership by CDP and ICLEI—Local Governments for Sustainability (CDP 2023). An open database such as the CDP with self‐submitted reports allows opportunities for critique, with the explicit goal of strengthening how future assessments are conducted. Selection criteria are shown in Figure 1 using a PRISMA flow diagram to ensure transparency (Page et al. 2021). The assessments were filtered to the publicly accessible reports submitted between 2018 and 2022 in the English language. This criterion ensures replicability of the review and that we were reviewing the most recent reports that were available at the time. Filtering for the English language was necessary for our understanding; however, it did limit international representation. We also excluded reports that were deemed to be out of scope, such as those exclusively focusing on mitigation. Of the 295 reports across 196 cities that met our criteria, we selected 80 cities for review using a simple random sampling method (see Figure 1).

In the next stage, we investigated which countries have Indigenous populations through the Native Land Map and population statistics available online through government websites (Native Land Digital 2025). This left 60 reports across 58 cities and regions, which were further purposively sampled by countries that had more than three reports for a representative sample size (Campbell et al. 2020; Palinkas et al. 2015), leaving 45 reports from cities and regions in four nations: the USA (26), Canada (10), Australia (5), and New Zealand (NZ) (4) (see Appendix 1). Historically, these nations were primarily impacted by British colonial forces, and Indigenous populations across these often have similarly fraught relationship dynamics with contemporary government institutions, leading to severely impacted inclusion in decision‐making (Schayegh 2024; Whyte 2016; Wolfe 2006). Similarities in these countries through linguistic accessibility, historical and embedded colonial contexts, policy frameworks and governance/institutional systems, and Indigenous rights movements allow for comparison of potentially similar treatment of Indigenous communities in CRAs and adaptation plans that may not be reflective of Indigenous populations in other countries with different historical and sociopolitical contexts. Nevertheless, the wider points of community inclusion in risk assessment processes and multiple worldviews for frameworks are relevant for all countries to consider for a diversity of perspectives and collaboration for climate adaptation planning.

During the initial stage, the 45 reports were examined by the primary author using summative qualitative content analysis (Hsieh and Shannon 2005) to identify the presence, absence, and basic forms of Indigenous recognition within CRAs by first conducting a keyword search (indigen, native, nation, tribe/al, tradition/al, local knowledge, first nation) and then reading report sections referencing “community,” “population,” or similar notions of public involvement. More thorough reading was undertaken as some reports mentioned “Indigenous” populations in their definitions of “frontline” or “vulnerable” communities, then referred to them by these latter terms throughout the report, whereas other CRAs used specific terminology (e.g., iwi (tribe/s) in Auckland (NZ) (Auckland Council 2019)), or the exact name of the group themselves (e.g., Lenape in Bethlehem (USA) (City of Bethlehem 2021)), and references may have otherwise been missed. It is significant to note that Indigenous communities are non‐homogenous, and representation in international CRAs could look very different depending on the population (Kelman et al. 2012).

In the second stage, reports that included Indigenous populations were examined for the extent to which Indigenous knowledge systems and values were recognized and included and if Indigenous connection to land was acknowledged, integrated, or ignored. The reports were initially placed into three categories using classic deductive thematic coding (Braun and Clarke 2006) through the assumption there would be no inclusion, some inclusion, and complete inclusion of Indigenous communities; however, this was expanded to five categories (see Table A1):

1. Complete lack of inclusion with Indigenous communities not mentioned at all.
2. Inclusion in a broad vulnerable or marginalized category.
3. The existence of Indigenous populations mentioned.
4. Indigenous communities given specific consideration with varying levels of recognition.
5. Varying inclusion of Indigenous worldviews and values.

Further themes of tokenism, future intentions, and recognition for the source of disproportionate risks emerged during the initial content analysis and keyword search, and were formally inductively analyzed after the categorization stage. All coding and analysis were conducted by the primary author to ensure consistency across documents. Analytic decisions and theme interpretations were iteratively and reflexively reviewed through engagement with co‐author expertise and relevant literature to strengthen rigor.

## 3 Results

Our review of city‐level CRAs revealed limited and inconsistent inclusion of Indigenous communities and perspectives. Most CRAs framed risk primarily through a technocratic lens, with only partial consideration of social and cultural factors. Where sociocultural aspects were acknowledged, engagement with local communities was uneven, and Indigenous communities, often those most affected by climate change, were the least consistently represented. The results are given through the five categories shared in Section 2.

### 3.1 Lacking Recognition of Indigenous Perspectives

**FIGURE 2:**
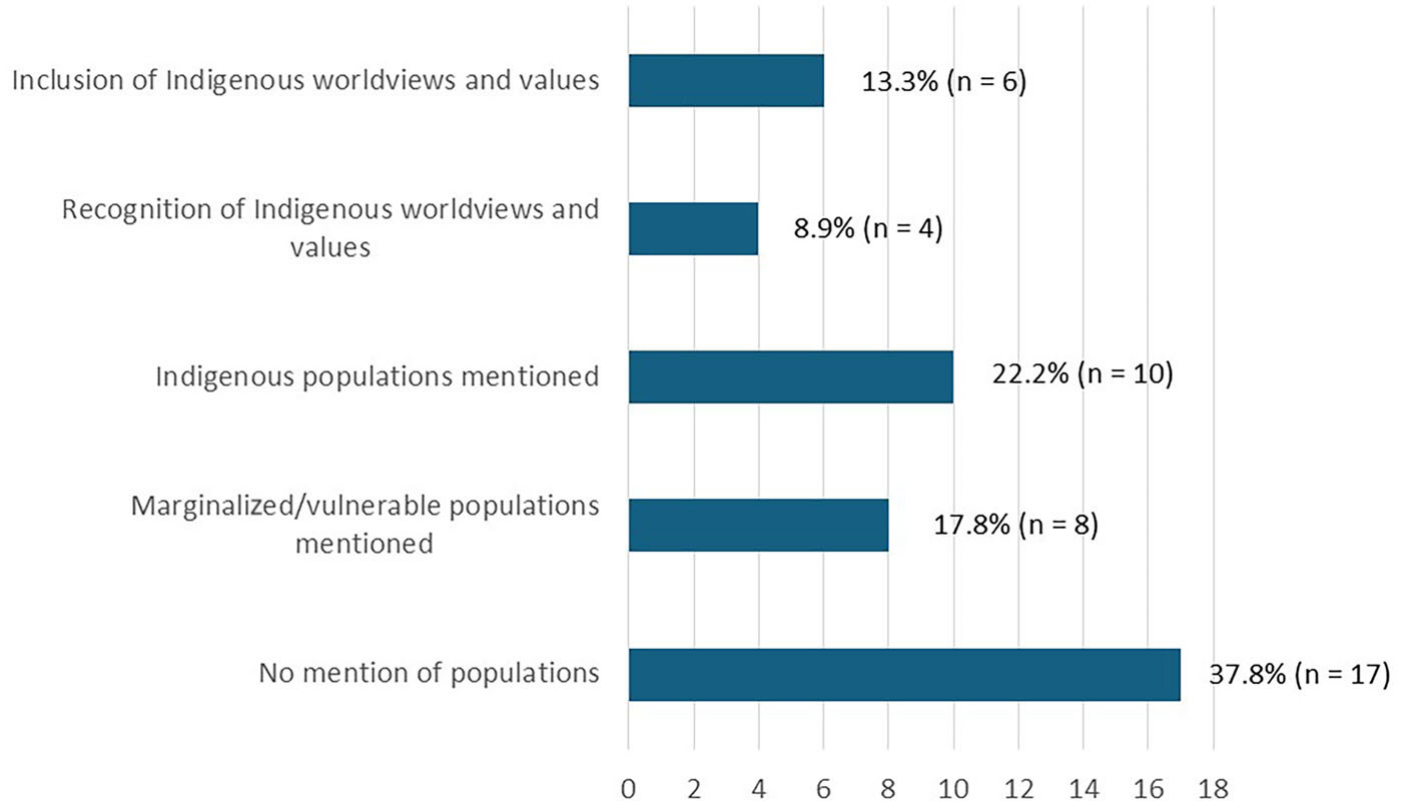
Bar chart showing the majority of the 45 reviewed CRA reports lack Indigenous community representation.

Content analysis of the 45 assessments revealed the extent of Indigenous representation (see Figure 2 and Table A1). 78% (35) of city‐level CRAs made little or no reference to Indigenous communities (Categories 1, 2, and 3 presented in Section 2). Some of these identified Indigenous peoples only as a vulnerable group, with minimal discussion of their distinct perspectives, priorities, or agency in shaping adaptation (Categories 2 and 3). Inductive thematic analysis also identified broader patterns and themes, including tokenistic representation of Indigenous communities, stated intentions of future engagement and collaboration with Indigenous communities often due to a lack of existing relationships, and a lack of recognition for the source of disproportionate risks to Indigenous communities.

Overall, the CRAs lacked sociocultural aspects and engagement with local communities (see Figure 2 and Table A1). Although some reports engaged communities in general, Indigenous populations were rarely consulted or engaged, despite being frequently recognized as more vulnerable than the general population. A total of 17 of the 45 reports (37.8%) did not mention Indigenous populations at all; however, this includes places with smaller Indigenous populations (<2%) and reports with no population disaggregation. A further eight reports (17.8%) referred only to “marginalized” or “vulnerable” communities, without specific consideration of Indigenous communities, who are often prescribed as “vulnerable” (IPCC 2023). Of the remaining 20 (44.4%) reports, 10 (22.2%) mentioned Indigenous communities only superficially, such as in population statistics or stated as specifically vulnerable. This included two Australian cities, Hobart and the Australian Capital Territory (ACT), that mentioned specific Indigenous populations in their Land Acknowledgments and referenced their ancestral connection to the environment, yet inclusion did not extend beyond the acknowledgment.

### 3.2 Representation of Indigenous Worldviews and Values

Only 10 of the 45 CRAs (22.2%) recognized or included Indigenous worldviews or culturally specific values (Categories 4 and 5, see Figure 2 and Table A1). Despite documented impacts of climate change to Indigenous identities and well‐being, many CRAs did not recognize Indigenous knowledge systems and place‐based values. Of those that recognized the necessity of Indigenous values, Auckland (NZ), stated:

It is crucial that [solutions around specific climate challenges] meet the specific needs and interests of our Māori communities, give a voice to our future generations through the work we do today, and give agency to the non‐human elements around us that make up the world that we are a part of. (Auckland Council 2019, 8)

Of the 10 (22.2%) reports that had some level of recognition or inclusion, five discussed intergenerational connections to the environment (see Figure 2 and Table A1). For example, Flagstaff (USA) identified culturally significant values and practices, including water, horses, and ceremonial sites, and assessed impacts to these for the Hopi Tribe, Navajo Nation, and others (City of Flagstaff 2018). Honolulu (USA) consistently stated that environmental impacts would disrupt Native Hawaiian customary practices due to their reliance on the collection of plants, animals, and minerals and the connection the environment has for Native Hawaiian and Polynesian identities (Climate Ready O'ahu 2020). Byron Shire in Australia recognized that “communities exist within human‐natural (or socio‐ecological) systems” (OEH 2019, 33), and Superior (USA) expanded upon this, stating “that humans are part of the ecosystem and that we are deeply connected to and reliant on the natural environment” (Boulder OEM 2022, 8) and that “the entire web of life and the inner connectivity and interactions are important” (Boulder OEM 2022, 45). Additionally, New Plymouth (NZ) noted that “the natural environment is at the heart of the nation's identity” and that disruption of these bonds, through adaptation actions such as relocation of communities, could cause grief, loss, and anxiety (Macara et al. 2022, 125).

In the 10 reports (22.2%) that recognized the necessity of Indigenous values, the authors frequently acknowledged that further work was required to achieve effective inclusion. Some reports (e.g., Dunedin, Vancouver, Saanich, and Bethlehem) expressed intentions to increase engagement and collaboration with “the public,” sometimes explicitly including Indigenous communities. Others (e.g., Byron Shire and New Plymouth) recognized that relationships needed to be established or strengthened before meaningful climate work could commence. Explanations for limited engagement included time, resourcing, capacity constraints, or lack of existing or well‐maintained relationships between government institutions and Indigenous communities. Byron Shire (Australia) discussed the need for value inclusion but recognized that there were “systemic difficulties engaging with Aboriginal communities,” noting that “knowledge management and access are not well connected,” which is compounded by government often having limited knowledge of Aboriginal culture (OEH 2019, 7–8). New Plymouth (NZ) stated their commitment to working with iwi (tribal groups) but recognized that their current plan “does not provide full details of how this commitment will be actioned due to the new relationship with various iwi groups being in very early stages.” (Macara et al. 2022, 14–15).

The Otago regional report submitted by Dunedin (NZ) was externally reviewed by the Indigenous service, Aukaha (2025), which found that the CRA methodology was limited by a lack of Māori (Indigenous to NZ) region‐specific knowledge, values, and practices. The report stated that their CRA did not reflect a Māori worldview and discussed commitment to future collaboration with Indigenous leadership groups, which “could” resemble a parallel assessment created by Māori, for Māori, framed by local knowledge systems and values (Otago Regional Council 2021, 16). Similarly, Bethlehem (USA) emphasized environmental justice for frontline communities, which included Indigenous communities, to “speak for themselves and respect lived experience as expertise,” noting that the creation of their Climate and Environmental Justice Plan “must be done in partnership with these communities” (City of Bethlehem 2021, 64). Byron Shire recognized that supporting Aboriginal cultural heritage and adaptation practices would assist their transition into a “transformed system” where “Aboriginal cultural heritage and voices are valued, considered, and embedded across all government service delivery areas” and knowledge and “connection to Country” are “incorporated in landscape management and recognized in legislation.” They state this level of inclusion would support autonomy and decision‐making (OEH 2019, 7–8).

Beyond the four cities that recognized the significance of Indigenous views and values, six reports (13.3%) demonstrated greater, though uneven, inclusion of Indigenous perspectives (see Figure 2 and Table A1). For example, Chicago was categorized as a city with inclusion of Indigenous values (in Table A1), yet beyond naming Indigenous Nations in the land acknowledgment, subsequent inclusion was framed under the term “frontline communities,” alongside wider communities of color and low‐income communities, who “experience the most immediate and worst impacts of climate change” due to factors that include “environmental challenges, and limited ability to influence decision‐making processes” (City of Chicago 2022, 6). Combining Indigenous communities alongside wider diverse groups into “frontline communities” limits the perspectives present and assumes common inequalities across groups, potentially limiting CRA effectiveness.

Other cities showed more consideration for the environmental concerns and cultural impacts on the local Indigenous communities (e.g., Honolulu (USA) and Flagstaff (USA)) and assessed impacts to natural and cultural resources, and some had more collaborative engagement (e.g., Saanich/British Columbia (Canada)) or future plans to do so (e.g., Auckland (NZ) and Taranaki (NZ)). Notably, none of these six reports contained specific Indigenous sociocultural hazard and risk framings.

### 3.3 Recognition for the Underlying Issues Exacerbating Disproportionate Risk to Indigenous Communities

**FIGURE 3:**
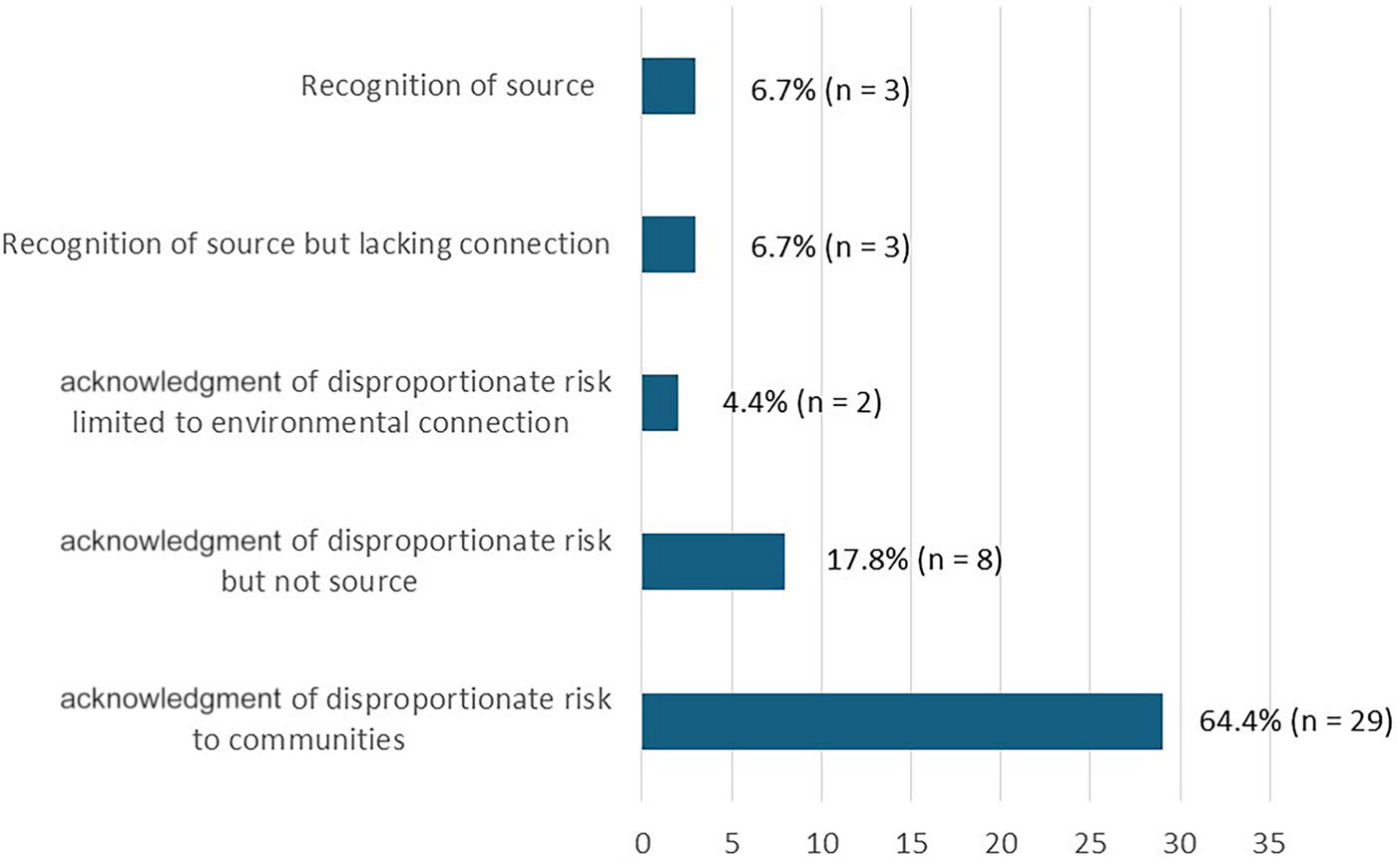
Bar chart showing that the majority of the 45 CRA reports did not recognize the source of disproportionate risk to Indigenous communities.

Another significant finding was that although Indigenous communities were acknowledged to be at disproportionate risk from climate change, most reports did not discuss why this was or how addressing these origins could contribute to climate justice. Only 16 of the 45 reports (35.5%) identified Indigenous communities as disproportionately at risk to climate change impacts (see Figure 3 and Table A2). Overall, 17.8% (8 of 45) reports acknowledged “race” as a contributing factor, alongside wider systemic conditions including socioeconomic status, access to health and education services, transportation, and language barriers. However, these reports did not address how these differential statuses arose, why this caused disproportionate consequences from climate risks, or the significance of historical and embedded inequities.

These eight reports (17.8%) often used language that passively attributed disproportionate risk to the impacted population themselves. For example, Cleveland (USA) noted that race and ethnicity, including being Indigenous, “strongly correlate with disparities in health, exposure to environmental pollution, and vulnerability to natural hazards, including climate‐related natural hazards” but then described a “tendency for non‐White populations to live closer to noxious facilities,” implying choice rather than structural drivers (City of Cleveland 2018, 20). Similarly, Bloomington (USA) stated that “people of color and limited English populations […] are at increased risk of exposure given their higher likelihood of living in risk‐prone areas” (City of Bloomington 2020, 72). Only two reports (4.4%), from Flagstaff (USA) and Honolulu (USA), linked disproportionate risk to environmental connection; however, even these did not engage with the structural sources of disproportionate risk arising from colonization and related systemic inequities.

Six reports (13.4%) recognized how climate risks were exacerbated for Indigenous communities through the legacies of colonization and discussed how this could be reflected in assessments and adaptation planning (see Figure 3 and Table A2). One of these reports (Medford (USA)) did not explicitly reference Indigenous communities due to the recorded Indigenous population being under 1%, instead attributing the source of disproportionate vulnerabilities to:

long‐standing patterns of poverty, systemic racism, segregation, gentrification, lack of political representation, weak or non‐existent social networks and connections, physical limitations, and quality of built infrastructure around people (City of Medford 2019).

Medford also noted that understanding how these structural patterns affect people is necessary for collaboration and climate resilience (City of Medford 2019, 58). Two regional reports (4.4%) from Dunedin/Otago (NZ) and Saanich/British Columbia (Canada) similarly emphasized the need for ongoing partnership and relationship development with Indigenous communities.

Chicago (USA) recognized the source of disproportionate risks, framing the assessment through a climate justice lens, which acknowledges “disparities and prioritizes the approaches that best address the needs of underserved communities” (City of Chicago 2022, 14). It recognized that “typically communities of color, Indigenous communities, and low‐income communities” faced “weakened community resilience due to compounding economic, health, and environmental challenges and limited ability to influence decision‐making processes,” noting some but still lacking recognition of deeper historical and financial inequities (City of Chicago 2022, 6). The report further stated that governments “must invest in climate actions that address and prevent furthering the legacy of social injustices” (City of Chicago 2022, vi).

This justice lens is amplified in the Superior (USA) report, which explicitly recognized the origins of disproportionate risks, detailing that:

Hazards do not impact all people equally. Climate change is a result of extraction, extraction of natural resources but also of people. The United States is built on the acceptability of extracting from Black and Indigenous people for the benefit of White male landowners including theft of land, slavery, and genocide. BIPOC have faced centuries of racism and discrimination that has been institutionalized into policies and practices at all levels of government and into our economic system. Social considerations are critical when assessing hazard impacts and identifying mitigation strategies that acknowledge inequities and shift towards a targeted universalism approach. (Boulder OEM 2022, 44)

Furthermore, Boulder noted that:

Despite many existing community strengths, disparities in income, racial equity, and lack of resource access create disproportionate vulnerabilities for some populations (Boulder OEM 2022, 149).

Similarly, Bethlehem (USA) specified that addressing historic environmental injustice and racism is necessary for resilience (City of Bethlehem 2021). However, the cities of Superior and Bethlehem were exceptions for consideration of community needs and application of a justice and equity lens. Overall, the findings reveal a lack of consistent sociocultural considerations for Indigenous worldviews and values and inclusion of environmental justice considerations.

## 4 Discussion

The limited and inconsistent inclusion of Indigenous perspectives in the 45 CRAs reflects deeper structural tendencies in how risk is framed and assessed. These findings relate to how Indigenous perspectives are represented within formal CRAs, rather than the full extent of engagement that may occur through other processes. Across the assessments analyzed, we found substantial variation in the depth and form of Indigenous inclusion, with most reports relying on technocratic or equity‐based framings that did not engage Indigenous perspectives as distinct ways of conceptualizing risk. As prevailing approaches privilege quantifiable dimensions of risk over sociocultural contexts and value systems, CRAs risk overlooking locally salient threats and advancing adaptation strategies that lack cultural legitimacy and effectiveness.

### 4.1 Limitations of Current Frameworks

Our analysis showed that current risk assessment frameworks remain narrowly technocratic, with limited incorporation of Indigenous perspectives, beyond token recognition. Such limited inclusion threatens overlooking culturally salient risks and undermines equitable and effective adaptation strategies.

A singular framing of risk, reflected in the limited recognition of Indigenous perspectives in the CRAs, excludes the diversity of perspectives present within countries and communities. Including diverse perspectives improves problem‐solving (Hong and Page 2004), reduces the likelihood of surprises (Dorji et al. 2024; Streets and Glantz 2000; Vaughan 1995; Vaughan and Seifert 1992), and supports more locally relevant framing of risks (Lazrus 2015; Le Heron et al. 2024; McNeeley and Lazrus 2014). Encompassing this diversity requires considering worldviews beyond dominant paradigms and political structures throughout the CRA process (Beck 2010; Lazrus 2015; Slimak and Dietz 2006; Vaughan and Seifert 1992), producing more widely accepted and legitimate outcomes (Blackett et al. 2024).

Our findings align with prior research on the marginalization of Indigenous knowledge in environmental governance and climate risk management (Dorji et al. 2024; Kelman et al. 2012; Whyte 2016). The majority of CRAs, 55.6% (25 of 45 reports), did not recognize Indigenous groups at all, and two referenced specific groups in land acknowledgments but made no further mention of these communities. Acknowledging ancestral land connections without follow‐through in a document assessing climate risks at a local level suggests a tokenistic form of inclusion. Although engagement may be occurring through other processes, we also argue that people should be included in CRAs regardless of where related equity information and processes are stored. Our findings support and add nuance to critiques that Indigenous perspectives are included across various documentation in tokenistic but not substantive ways (Maxwell et al. 2020; Smith 2022), despite the relevance of Indigenous worldviews and values for analyzing risk.

Across the CRAs reviewed, the omission of cultural dimensions was associated with weaker risk communication, as reports failed to engage meanings, priorities, and concerns salient to Indigenous communities. Risk communication grounded solely in technocratic approaches is less likely to resonate, as cultural norms, narratives, and values shape how information is interpreted (Vaughan 1995; Adger et al. 2012; Hansson 2009). Dialogic and participatory approaches can improve relevance and legitimacy by engaging communities’ lived experiences and value systems (Jardine 2008; Rich et al. 1995).

Many CRAs also failed to address the historical and embedded inequities contributing to Indigenous exposure to climate risk, rarely recognizing that colonization and systemic disadvantage have shaped place and socioeconomic conditions (Barnett et al. 2023; Whyte 2016). Some reports passively attributed disproportionate risk to the “tendencies” of impacted communities to live in hazard‐prone areas, obscuring how polluting sites were historically sited near marginalized communities with impacted political power (Logan et al. 2021, 5; Nettler 2022; Northern 1997). Attributing increased vulnerability to dependence on local ecosystems frames risk as a matter of choice, obscuring that all societies depend on these systems (Whyte 2016, 13). As a result, Indigenous and similarly marginalized communities often experience increased sensitivity and exposure to climate change through indirect risk pathways shaped by historical inequities that are rarely captured in CRAs (Anderson et al. 2024; Barnett et al. 2023). Ignoring these origins threatens to reproduce inequities in future adaptation; addressing environmental justice, therefore, requires addressing the colonial structures and socioeconomic drivers that restrict Indigenous environmental care and self‐determination (Whyte 2016; Veland et al. 2013).

Furthermore, a lack of robust relationships between CRA practitioners and Indigenous communities limits meaningful engagement. Although 22.2% (10 of 45) reports expressed intentions for future collaboration, they often cited time and resource constraints or cultural knowledge gaps within government agencies. Future research could determine if this intention goes beyond well‐meaning sentiment. Building relationships on consent, trust, accountability, and reciprocity takes time and effort (Whyte 2020, 2), exemplifying the need for parallel Indigenous‐led assessments or frameworks designed to incorporate multiple worldviews (Nadasdy 1999).

### 4.2 Why Inclusion of Worldviews and Values Matters

The findings demonstrate that Indigenous worldviews and values are rarely incorporated into the conceptual foundations of CRAs, even where Indigenous communities are acknowledged. Inclusion of worldviews and values was inconsistent or entirely lacking. Differences in sociocultural constructions of risk must be understood and included throughout the risk management process (Slimak and Dietz 2006; Thompson and Wildavsky 1982). Conflicting views between practitioners and communities reflect differences in values as well as knowledge, which must be incorporated into CRAs (Pidgeon 1998; Slovic 1987, 2020). These value differences can cause conflicts or inconsistencies between adaptation options deemed acceptable by practitioners and those accepted by individuals and communities, affecting support and motivation to respond as well as the validity of the CRA itself (Adger et al. 2012; McComas et al. 2008).

Lacking a range of views in decision‐making processes is a substantial barrier to adequately incorporating value systems across multiple scales (Adger et al. 2012). Given the patterns of limited and tokenistic inclusion identified in this study, the absence of collaborative and dialogic engagement with Indigenous communities represents a potential risk to the applicability and effectiveness of CRAs. Collaborative risk reduction approaches that engage local concerns and reflect on the politics, ethics, and moral implications of who is excluded from decision‐making are, therefore, needed (Adam et al. 2000; Beck 1996). As those who control the “definition” of what is measured as risk also control the solutions, “defining risk is thus an exercise in power” where the power dynamics within a country's governance structure shapes how options for risk management are prioritized (Slovic 1998, 76). This is evident in the power dynamics between risk assessors and the Indigenous populations they claim to include in their CRAs in the vast majority of CRAs studied. That said, it is difficult to adequately represent varying value systems in a singular framing of climate risk (Pidgeon 1998; Otway and Thomas 1982), lending credence to the notion of multiple frameworks or parallel Indigenous‐led assessments.

### 4.3 Consequences of Excluding Indigenous Worldviews

The consequences of excluding Indigenous worldviews and values from CRAs can be understood through normative, instrumental, and substantive dimensions (Balog‐Way et al. 2020; Demeritt and Nobert 2014; Rickard 2021; Wardman 2008). These include failing to capture diverse perspectives needed to reduce surprise and maladaptation (Barnett and O'Neill 2013; Dorji et al. 2024; Shah et al. 2025; Streets and Glantz 2000; Vaughan 1995); overlooking risks to what Indigenous communities value (McComas et al. 2008; Slovic 1999; Wale and Huson 2024), and missing opportunities to co‐develop ecosystem‐based adaptation strategies (Blackett et al. 2024; Whyte 2016). Neglecting Indigenous worldviews and values ignores interconnected place‐based frameworks and a range of risks and adaptation options that would not be identified otherwise.

Indigenous concerns extend beyond the environment, and Indigenous agency holds significant value in governance and related systems (Barnett et al. 2023). When Indigenous communities are subsumed within broader environmental justice or “frontline community” framings, such as in Chicago, CRAs may advance equity goals while failing to fully engage Indigenous values as distinct epistemological and relational foundations for understanding risk. Indigenous cultures’ long‐standing relationships with the natural environment emphasize human–ecosystem interconnections that shape identities, social networks, sense of place, and intergenerational knowledge systems (Adger et al. 2012; Durie 2005; Menzies et al. 2022; Royal 2003; Wickham et al. 2022; Yletyinen et al. 2022). These reciprocal, place‐based relationships significantly influence risk perceptions and prioritize ecosystem‐based adaptation options for specific local contexts that are often unsupported by prevailing technocratic CRA frameworks (Bailey‐Winiata et al. 2024; Harmsworth and Raynor 2006; Whaanga et al. 2017).

Indigenous communities are diverse and non‐homogenous, requiring context‐specific representation in CRAs (Kelman et al. 2012). However, there are similarities in the ways Indigenous communities understand risk based on their relationships to their environments (Harmsworth and Raynor 2006). For example, the Māori worldview in NZ is described as providing ecosystem‐based solutions for climate risk management (Blackett et al. 2024; Hyslop et al. 2023), and Anishinaabe in Canada highlight that no single climate impact can be prioritized when the environment and ways of life are interconnected (Menzies et al. 2022). Similar place‐based values and impacts to them from climate change can be seen across Indigenous communities internationally, including in Canada (Bertolas 1998; Menzies et al. 2022; Wale and Huson 2024), the United States (Rosales and Chapman 2015), Australia (Barnett et al. 2023; Nash et al. 2018; Petheram et al. 2010), NZ (Bailey‐Winiata et al. 2024; Hikuroa 2022), Siberia (Crate 2008), and the Solomon Islands (Butcher 2019), among many others. Although awareness of the significance of Indigenous risk perceptions and holistic worldviews for approaching complex risks is growing (Ihirangi 2021; IRGC 2017), our review shows that many CRAs lack meaningful incorporation of Indigenous worldviews and values. Excluding Indigenous worldviews and place‐based knowledge systems limits adaptation options, does not represent community interests and priorities, and may contribute to the rejection of some risk management decisions (de Goër de Herve et al. 2023; Lazrus 2015; McComas et al. 2008; McNeeley and Lazrus 2014).

### 4.4 Toward Pluralistic Assessments and Multiple Frameworks

Limited inclusion of Indigenous communities in the CRAs analyzed perpetuates singular technocratic approaches and top‐down communication, rather than co‐producing culturally relevant adaptation strategies. Reflecting diverse viewpoints can help establish common ground and support self‐determination (Blackett et al. 2024; Kelman et al. 2012; Pidgeon 1998). The aim is not to establish one viewpoint but to examine the circumstances shaping decision‐making within climate adaptation so actions are effective and relevant for the affected communities (Veland et al. 2013). Although CRA practitioners largely default to technocratic frameworks, our results indicate emerging intentions for more substantive engagement with Indigenous communities or to provide space for parallel Indigenous‐framed and led assessments, such as suggested by Dunedin (NZ) (Otago Regional Council 2021). Agency is critical to the effectiveness of cultural impact assessments, with those led *by* Indigenous communities, *for* Indigenous communities, being more representative of Indigenous priorities, though wider structural factors could still limit agency and effectiveness of the outcomes (Jolly and Thompson‐Fawcett 2023). The prevailing paradigm formed from historical and embedded colonial structures within certain countries, including those discussed here, has caused inequitable decision‐making abilities (Whyte 2016). The resulting power dynamics and systemic inequities impact how and to what extent CRAs engage with Indigenous communities and their values.

Although this study identifies recurring patterns of exclusion or limited inclusion across CRA documents, the content analysis approach adopted cannot explain how internal institutional processes, resource allocation, or governance arrangements shape these outcomes. Further research is required to determine how Indigenous worldviews can be collaboratively brought into current CRA frameworks or how parallel assessments from Indigenous frameworks can be appropriately operationalized given the need for intersectionality and agreement on certain adaptation options. Indigenous communities perceive risk through holistic, interconnected lenses that include past experiences and ancestral connections to the environment and transmission of knowledge and cultural practices, all of which is necessary to encompass in risk assessments (Bailey‐Winiata et al. 2024; Blackett et al. 2024; Lazrus 2015). Future research must consider how Indigenous‐led assessments are framed, how risk is understood, and the extent to which community‐led, driven, and maintained risk management makes a difference to community support, uptake of actions, and adaptation outcomes. Bridging the relational theory of worldviews and values constructing risk with the practicalities of CRAs could suggest how multiple CRA frameworks might coexist, with overlapping assessments addressing, at minimum, how communities rely on each other for economic, infrastructural, sociocultural, and other types of support. Although these understandings of risk will often be complementary, there will sometimes be contradictions (Boholm and Corvellec 2011), which presents challenges for decision‐making but will inevitably improve CRAs. Future reflection on the assessments reviewed would provide insight into how these cities actioned their intentions and how parallel Indigenous‐led CRAs could be conducted.

### 4.5 Limitations of This Research

Due to the ongoing development of CRA practices, more recent or unpublished approaches are not captured here. Nevertheless, this review provides a comprehensive overview of how Indigenous communities are currently represented in global CRAs. Limitations arise from reliance on reports self‐submitted to the CDP database, resulting in inconsistency in what was considered a risk and vulnerability assessment and scalar variation, including submissions at regional or country level. As cities submitted reports relevant to the areas they inhabit, some assessments were conducted at a regional scale, meaning the location assessed may be larger than the named reporting city (see Appendix 1). Nevertheless, all CRAs analyzed here focus specifically on climate risks and vulnerabilities. Additional research into the institutional structures requesting and conducting these reports, including in‐depth interviews with practitioners, would further support CRA development.

There is also variability across reports as units of analysis; limiting direct comparability due to differences across scale; Indigenous population size, access to resources to conduct CRAs, which can shape depth, scope, and quality; and inconsistent reporting requirements across and within countries. Rather than compare reports, this analysis examines how Indigenous perspectives are represented in formal CRAs, given the relevance of multiculturalism in statutory requirements (Anaya 2015; Jacobson et al. 2016; Sanders 1983; Wensing 2018), environmental justice (Whyte 2016; Veland et al. 2013), and diverse risk framings, worldviews, and place‐based knowledge systems for locally specific climate action (de Goër de Herve et al. 2023; Wickham et al. 2022).

As this review examines only evidence self‐submitted to the CDP, it does not capture how cities engaged with Indigenous communities beyond the CRAs. Future research could examine wider engagement processes through interviews with assessment practitioners and local council members. The lack of research on wider municipal activities limits result analysis, as Indigenous populations were often smaller in cities and, therefore, less represented than in regional assessments. Indigenous communities also extend beyond city boundaries, with contested land rights adding further complexity. Further research on regional‐level assessments would contribute to understanding the significance of Indigenous frameworks for environmental management in climate change.

## 5 Conclusion

Current CRAs across the United States, Canada, Australia, and New Zealand show deficient consideration for multiple worldviews and value systems, despite the significance of Indigenous communities. Risk analysis literature demonstrates that worldviews and values are fundamental to understanding what is at risk and how to respond (Lazrus 2015; Pidgeon 1998) and highlights the importance of Indigenous knowledge systems for holistic and just risk management (de Goër de Herve et al. 2023; Wale and Huson 2024; Whyte 2016; Veland et al. 2013). Our findings show that Indigenous inclusion is often absent, superficial, or insufficient to structurally influence how risks are defined and assessed. Taken together, these findings indicate that current CRAs do not simply omit Indigenous perspectives but constrain how risk is defined and evaluated in practice. This has both normative and practical implications. Excluding diverse cultural framings, worldviews, and values limits the scope of risks that are identified and prioritized and reflects broader power dynamics in global knowledge systems. Although some CRAs indicate emerging intentions to engage more meaningfully with Indigenous communities, these are not yet reflected in how risk is conceptualized within assessment frameworks. As a result, adaptation strategies may fail to prioritize what matters most to local communities, reducing their effectiveness and legitimacy.

CRAs in regions with Indigenous populations, therefore, require meaningful engagement with Indigenous perspectives, not only to improve equity but to enhance the quality and relevance of risk assessments. Indigenous framed or Indigenous‐led parallel assessments can provide space for decision‐making grounded in place‐based, intergenerational knowledge systems, offering alternative understandings of risk and pathways for adaptation not captured by prevailing technocratic approaches. Effectively including Indigenous worldviews and values requires collaboration with communities to examine exclusion through historical colonial processes and the resulting structural inequities in wider decision‐making. Recognizing and addressing the historical and structural drivers of disproportionate climate risk is a necessary step toward more effective and just adaptation.

Bringing Indigenous worldviews into CRAs requires approaches that account for how risk is constructed through relationships between natural phenomena, valued elements, and observers. Relational theories of risk (Boholm and Corvellec 2011) offer a promising conceptual basis for understanding these dynamics, although further research is needed to operationalize such approaches in practice. Moving beyond tokenistic inclusion and singular technocratic frameworks toward pluralistic, participatory, and dialogic approaches to risk assessment is essential. Integrating Indigenous worldviews and knowledge systems within, or alongside, existing frameworks can support more adaptive, culturally grounded, and effective responses to climate risks.

## Funding

T.L. and M.S. received funding from MBIE Smart Idea: Innovating climate risk assessment: A system‐wide, geospatial approach for councils and communities. T.L. received Rutherford Discovery Fellowship: Incorporating cascading risk and multiple uncertainties into climate adaptation planning. European Union's Horizon Europe research and innovation programme: grant agreement number 101147385.

## Conflicts of Interest

T.L. has interests in the risk company Urban Intelligence and its software Resilience Explorer.

## Data Availability

The data that support the findings of this study are available from the corresponding author upon reasonable request.

## Appendix 1

### 1.1

**Table:**

City	Location of report	Country	Year	Plan title
Alameda, CA	Alameda	United States	2019	Alameda Climate Action and Resiliency Plan
Ajax, ON	Ajax	Canada	2019	Ajax Climate Risk and Resiliency Plan
Asheville	Asheville	United States	2018	Planning for Climate Resilience City of Asheville, North Carolina
Atlanta, GA	Atlanta metropolitan area/region	United States	2018	Vulnerability and Resiliency Framework for the Atlanta Region
Auckland	Auckland	New Zealand	2019	Climate change risks in Auckland
Austin, TX	Austin	United States	2018	Climate Resilience Action Plan for City Assets and Operations
Australian Capital Territory, ACT	Australian Capital Territory	Australia	2022	Climate change risk assessment for the ACT
Bethlehem, PA	Bethlehem	United States	2021	City of Bethlehem, PA Climate Action Plan 2021
Bloomington, IN	Bloomington	United States	2020	Bloomington Climate Risk and Vulnerability Assessment May 2020.pdf
Boca Raton, FL	Southeast Palm Beach County	United States	2021	Multi‐jurisdictional Climate Change Vulnerability Assessment
Boynton Beach, FL	Southeast Palm Beach County	United States	2021	Multi‐jurisdictional Climate Change Vulnerability Assessment
Burlington, ON	Burlington	Canada	2021	Climate Resilient Burlington: Climate Change Vulnerability & Risk Assessment
Byron Shire, NSW	North Coast region	Australia	2019	North Coast Enabling Regional Adaptation: North Coast Region Report
Calgary, AB	Calgary	Canada	2021	The City of Calgary Community Climate Risk Index: Summary Report
Chicago, IL	Chicago	United States	2022	Chicago Climate Action Plan
Cleveland, OH	Cleveland	United States	2018	Cleveland Climate Action Plan: Climate and Social Vulnerability Assessment—Appendix C
Columbus, OH	Columbus	United States	2018	Columbus Climate Adaptation Plan
Duluth, MN	Duluth	United States	2018	Climate Vulnerability and Adaptation in Duluth, Minnesota
Dunedin	Otago region	New Zealand	2021	Otago Climate Change Risk Assessment
Edmonton, AB	Edmonton	Canada	2018	Climate Change Vulnerability and Risk Assessment for City of Edmonton
Fayetteville, AR	Fayetteville	United States	2018	2018 Fayetteville Climate Resilience Assessment
Flagstaff, AZ	Flagstaff	United States	2018	Flagstaff's Vulnerability to Climate Change
Fort Collins, CO	Fort Collins	United States	2019	2019 Municipal Sustainability and Adaptation Plan
Hamilton, ON	Hamilton	Canada	2021	City of Hamilton: Vulnerability & Risk Assessment Report
Hobart, TAS	Hobart	Australia	2020	Sustainable Hobart Action Plan
Honolulu, HI	City and County of Honolulu	United States	2020	Climate Ready Oʻahu Risk Assessment Results
Houston, TX	Houston	United States	2020	Climate Impact Assessment for the City of Houston
Kansas City, MO	Kansas City region	United States	2022	Climate Risk and Vulnerability Assessment
Knoxville, TN	Knox County	United States	2018	Multi‐Jurisdictional Local Hazard Mitigation Plan
Los Angeles, CA	Los Angeles	United States	2018	City of Los Angeles 2018 Local Hazard Mitigation Plan
Louisville, KY	Louisville	United States	2020	Climate Change Vulnerability in Louisville, Kentucky
Manhattan Beach, CA	Manhattan Beach	United States	2021	Sea Level Rise Risk, Hazards, and Vulnerability Assessment, City of Manhattan Beach
Medford, OR	Medford	United States	2019	Medford Climate Change Vulnerability Assessment
Miami, FL	Southeast Florida region	United States	2019	Unified Sea Level Rise Projection Southeast Florida
Mount Barker District, SA	Mount Barker District	Australia	2019	South Australia pilot climate change adaptation governance assessment: Climate change adaptation governance assessment report for Mount Barker District Council
Nanaimo, BC	Nanaimo	Canada	2020	Climate Change Resilience Strategy
New Plymouth	Taranaki region	New Zealand	2018	Civil Defense Emergency Management Group Plan for Taranaki 2018–2023
			2022	Climate change projections and impacts for Taranaki
Saanich, BC	British Columbia	Canada	2021	Climate Preparedness and Adaptation Strategy
			2019	Preliminary Strategic Climate Risk Assessment for British Columbia
San Francisco, CA	City and County of San Francisco	United States	2020	City and County of San Francisco Hazards and Climate Resilience Plan March 26, 2020
Saskatoon, SK	Saskatoon	Canada	2019	Climate change projections and possible impacts
St. Catharines, ON	St. Catharines	Canada	2021	Corporate climate change adaptation plan: Preparing for a changing future
Superior, CO	Boulder County	United States	2022	2022–2027 Boulder Hazard Mitigation Plan
Unley, SA	Unley	Australia	2021	Climate Change Adaptation Governance Assessment: Climate Change Adaptation Governance Assessment Report for the City of Unley
Vancouver, BC	Vancouver	Canada	2018	Climate Change Adaptation Strategy
Wellington	Wellington	New Zealand	2021	Coastal hazards and sea‐level rise in Wellington City

## Appendix 2

### 2.1

**TABLE A1:** Table showing the extent of Indigenous representation and value inclusion in the 45 climate risk assessment (CRA) reports.

Extent of inclusion	Number of reports	Names of reporting cities
No mention of populations	37.8% (*n* = 17)	Asheville (USA), Atlanta (USA), Austin (USA), Burlington (Canada), Columbus (USA), Edmonton (Canada), Fort Collins (USA), Hamilton (Canada), Houston (USA), Knoxville (USA), Louisville (USA), Miami (USA), Mt Barker (Australia), Saskatoon (Canada), St Catharines (Canada), Unley (Australia), Wellington (NZ)
Marginalized/vulnerable populations acknowledged	17.8% (*n* = 8)	Alameda (USA), Ajax (Canada), Boynton (USA), Boca Raton (USA), Fayetteville (USA), Manhattan Beach (USA), Medford (USA), Kansas City (USA)
Indigenous populations mentioned	22.2% (*n* = 10)	Australian Capital Territory (ACT) (Australia), Bloomington (USA), Calgary (Canada), Cleveland (USA), Duluth (USA), Hobart (Australia), Los Angeles (USA), Nanaimo (Canada), San Francisco (USA), Vancouver (Canada)
Recognition of Indigenous worldviews and values	8.9% (*n* = 4)	Auckland (NZ), Byron Shire (Australia), New Plymouth (NZ), Saanich (Canada)
Inclusion of Indigenous worldviews and values	13.3% (*n* = 6)	Bethlehem (USA), Chicago (USA), Dunedin (NZ), Flagstaff (USA), Honolulu (USA), Superior (USA)

## Appendix 3

### 3.1

**TABLE A2:** Table of reports that recognized the source of disproportionate risk for Indigenous communities.

Extent of disproportionate risk source acknowledgment	Number of reports	Names of reporting cities
No acknowledgment of disproportionate risk to communities	64.4% (*n* = 29)	Alameda (USA), Ajax (Canada), Asheville (USA), Atlanta (USA), Austin (USA), Australian Capital Territory (ACT) (Australia), Boynton (USA), Boca Raton (USA), Burlington (Canada), Columbus (USA), Edmonton (Canada), Fayetteville (USA), Fort Collins (USA), Hamilton (Canada), Hobart (Australia), Houston (USA), Kansas City (USA), Knoxville (USA), Los Angeles (USA), Louisville (USA), Manhattan Beach (USA), Miami (USA), Mt Barker (Australia), Nanaimo (Canada), New Plymouth (NZ), San Francisco (USA), Saskatoon (Canada), St Catharines (Canada), Unley (Australia), Wellington (NZ)
Acknowledgment of disproportionate risk but not source	17.8% (*n* = 8)	Auckland (NZ), Bloomington (US), Byron Shire (Australia), Calgary (Canada), Cleveland (USA), Duluth (USA), Manhattan Beach (USA), Vancouver (Canada),
Acknowledgment of disproportionate risks limited to environmental connection	4.4% (*n* = 2)	Flagstaff (USA), Honolulu (USA)
Recognition of source but lacking connection	6.7% (*n* = 3)	Chicago (USA), Dunedin (NZ), Saanich (Canada)
Recognition of source	6.7% (*n* = 3)	Bethlehem (USA), Medford (USA), Superior (USA)
